# Association of Poor Sanitation With Growth Measurements Among Children in India

**DOI:** 10.1001/jamanetworkopen.2020.2791

**Published:** 2020-04-15

**Authors:** Suman Chakrabarti, Parvati Singh, Tim Bruckner

**Affiliations:** 1 Institute for Health Metrics and Evaluation, Department of Health Metrics Sciences, University of Washington, Seattle; 2 Program in Public Health, Anteater Instruction and Research Offices, University of California, Irvine

## Abstract

**Importance:**

Much research on sanitation and linear growth in low- and middle-income countries focuses on children younger than 5 years. However, poor sanitation may be associated with growth faltering during middle and late childhood to a greater extent than previously recognized.

**Objective:**

To characterize the association of poor sanitation with height-for-age *z* (HAZ) scores in children and adolescents in India aged 0 to 18 years.

**Design, Setting, and Participants:**

This cross-sectional study examined 134 882 children and adolescents aged 0 to 18 years who were surveyed in the fourth round of India’s District Level Household and Facilities Survey (survey conducted August 2012 to February 2014). Data were analyzed from June 1, 2019, to August 20, 2019.

**Exposures:**

Proportion of households reporting open defecation at the village level (to account for its high negative externality) and household-level access to boiled or filtered drinking water.

**Main Outcomes and Measures:**

Individual-level HAZ scores were measured in standard deviations. The association of exposures with outcomes was estimated using ordinary least-squares regression stratified by sex (boys and girls) and 4 age groups (≤1, >1 to ≤7, >7 to ≤12, >12 to ≤18 years). Models controlled for parental height and education, socioeconomic status, maternal age at birth, hemoglobin level, and indicators for state and birth year to adjust for regional (state) and temporal (birth year) fixed effects.

**Results:**

The sample comprised 70 463 male (52.5%) and 64 419 female (47.8%) children and adolescents aged 0 to 18 years; 46 722 participants (34.6%) were aged older than 12 to 18 years. Open defecation was inversely associated with HAZ score among all age groups except boys aged 1 year and younger (>1 to ≤7 years: β, −0.22; 95% CI, −0.35 to −0.10; >7 to ≤12 years: β, −0.15; 95% CI, −0.24 to −0.06; >12 to ≤18 years: β, −0.10; 95% CI, −0.19 to −0.01) and among girls aged between 7 and 18 years (>7 to ≤12 years: β, −0.22; 95% CI, −0.33 to −0.12; >12 to ≤18 years: β, −0.16; 95% CI, −0.23 to −0.09). Boiled or filtered drinking water was positively associated with HAZ score among younger girls (≤1 year: β, 0.26; 95% CI, 0.07 to 0.45; >1 to ≤7 years: β, 0.07; 95% CI, 0.01 to 0.14) and across all age groups in boys (≤1 years: β, 0.19; 95% CI, 0.03 to 0.35; >1 to ≤7 years: β, 0.07; 95% CI, 0.00 to 0.14; >7 to ≤12 years: β, 0.08; 95% CI, 0.03 to 0.13; >12 to ≤18 years: β, 0.06; 95% CI, 0.01 to 0.11).

**Conclusions and Relevance:**

In this study, open defecation and lack of boiled or filtered drinking water were inversely associated with height-for-age measures across all ages in children and adolescents in India. Improved sanitation may benefit growth among children and adolescents older than 5 years.

## Key Points

**Question:**

Is poor sanitation associated with lower height among children older than 5 years in India?

**Findings:**

This cross-sectional analysis of 134 882 children and adolescents aged 0 to 18 years in India found that poor sanitation (ie, open defecation and lack of access to boiled or filtered drinking water) was associated with lower height-for-age *z* scores across all ages in childhood and adolescence.

**Meaning:**

Improved sanitation may be associated with greater height across all child ages.

## Teaser

This cross-sectional study characterizes the association of poor sanitation with height-for-age *z *scores in children and adolescents in India aged 0 to 18 years.

## Introduction

Low height-for-age, or anthropometric growth faltering in children, serves as a key indicator of chronic undernutrition.^1^ Growth failure in early childhood also reportedly diminishes growth potential during adolescence.^2,3^ For this reason, much research on undernutrition and child growth focuses on the first 1000 days of life and on children younger than 5 years.^1,4^ However, the most recent Global Nutrition Report^5^ underscores a potentially important role of height gain during later childhood in mitigating prior growth failure. Preadolescence and adolescence may serve as periods of compensatory growth and reduce the height gap observed between high-income and low- or middle-income countries (LMICs).^6,7^ The possibility of compensatory growth suggests that the current and exclusive focus on growth faltering among children younger than 5 years may need reevaluation.^7,8,9^

Much of Europe exhibited gains in height following increases in income (indicating nutritional intake) and improvements in health care, nutrition, and sanitation over the past 2 centuries.^10,11^ By contrast, among LMICs, height is not strongly associated with economic growth and development over time.^10^ In particular, India remains an outlier, with a high prevalence of stunted growth despite significant and sustained economic progress.^12^ Scholars contend that these trends in India derive from the practice of open defecation and poor sanitation.^13^ Studies show that at the same socioeconomic or income levels, children and adolescents in India who are exposed to ambient open defecation display greater stunting (ie, height-for-age *z* [HAZ] scores less than −2 SDs) than their unexposed counterparts.^14^ Open defecation carries high negative externality given that this behavior exposes individuals to fecal pathogens despite good personal sanitation habits.^15^ Contamination of environmental surroundings and drinking water with fecal pathogens increases disease burden and reduces nutrient absorption, leading to chronic undernutrition and stunting.^16,17^ As of 2012, nearly half of all households in India practiced open defecation^18^ and lacked access to safe drinking water.^19^ India currently accounts for almost one-third of the world’s stunting burden.^5^

Approximately 45% of adult bone mass and 20% of total height develops during adolescence.^20^ This developmental period may serve as the final window of opportunity to reduce stunting.^20^ Improvements in disease environment, sanitation, and resource availability may stimulate remedial growth in middle and later childhood. Clinical studies show that physiologic damage (eg, inflammation of intestinal tissue leading to poor nutrient absorption) from sustained exposure to open defecation and unclean drinking water appears reversible—within a relatively short period—upon relocation to clean settings.^21,22,23^ Children from LMICs, including India, who migrate to or are adopted in Europe and the United States exhibit rapid gains in HAZ score and attain greater than expected adult height relative to their native populations, despite growth retardation in infancy or early childhood.^20^ For instance, severely stunted girls from India who were adopted in Sweden gained a mean of 2 SDs in HAZ score by puberty.^24^ Whereas most of these adoptees showed early pubertal development (a consequence of early undernutrition), they also demonstrated considerable remedial growth.^25^ Taken together, these findings indicate plausible recovery from early nutritional insults and motivate the extension of research to children and adolescents older than 5 years. This issue holds particular salience in India because continued exposure to poor sanitation may accrue over time and exert an influence on growth faltering during later stages of childhood that is greater than what the literature reports.^26^

We know of no population-level study that examines the association of poor sanitation with child growth among children and adolescents older than 5 years in India. We used a large survey data set to examine whether and to what extent 2 correlates of ambient sanitation—open defecation and boiled or filtered drinking water—were associated with HAZ scores across multiple age groups in children and adolescents (ie, 0 to 18 years). We examined 4 age groups (≤1 year, >1 to ≤7 years, >7 to ≤12 years, and >12 to ≤18 years) to ascertain distinct associations of HAZ scores with open defecation and boiled or filtered drinking water, controlling for parental endowment (height, education, maternal age at birth), socioeconomic status (SES), and rural or urban area of residence. Given differential child growth patterns among boys and girls, we conducted separate descriptive and analytic exercises by sex. Our research, unlike prior work, describes the plausible association of poor sanitation with growth faltering over the entire span of childhood in India*.*

## Methods

### Study Population

We use deidentified, publicly available cross-sectional data for 134 882 children and adolescents aged 0 to 18 years in India from the fourth round of India’s District Level Household and Facilities Survey (DLHS-4).^27^ The District Level Household and Facilities Survey is a national health survey conducted in 23 Indian states that is sampled to be representative at district and state levels. This survey was conducted by the International Institute of Population Studies from August 2012 to February 2014.^27^ The University of California, Irvine, institutional review board deemed this study exempt owing to the use of publicly available, deidentified data. We followed the Strengthening the Reporting of Observational Studies in Epidemiology (STROBE) reporting guideline for this study.^28^

### Outcome Variable

We obtained the date of birth and date of survey (both in date/month/year format) from DLHS-4 and calculated age in months per child aged 0 to 18 years within surveyed households.^27^ We retrieved data on height (in centimeters) per child and formulated HAZ scores of children and adolescents aged 0 to 18 years as our outcome per World Health Organization growth standards using the *z-anthro* STATA routine.^29^ A detailed description of DLHS-4 child anthropometric measurement protocols appears in eAppendix 1 in the Supplement.

### Explanatory Variables

Our exposure variables, ie, open defecation and access to boiled or filtered drinking water, gauge poor sanitation. We derived village-level (and equivalent urban sampling clusters in cities) open defecation from the proportion of households reporting no access to a toilet facility in a village.^15,30^ This specification aligns with prior research that used open defecation as an ecological exposure.^14,15,30,31^ We defined access to boiled or filtered drinking water as a binary variable with households using boiling and/or water filtration systems (electric, ceramic, and composite material filters) coded as 1 and all others (indicating use of unclean drinking water) coded as 0. We used this specification (of clean drinking water) as opposed to the commonly used specification of improved sources of drinking water because the latter does not adequately reflect measures to remove microbial contamination.^32,33^ We also retrieved other covariates of interest, including parental height and education, household SES, place of residence (rural or urban, based on Census of India classifications^34^), and hemoglobin levels.^35,36,37,38^ We formulated the household SES variable using principal component analysis of household assets, including toilet facility, cooking fuel type, water source, dwelling type, access to electricity, and durable assets.^39^ We did not include diarrhea as a covariate because it falls on the causal pathway linking poor sanitation to stunting. Moreover, its record collection in DLHS-4 depends on occurrence within 2 weeks before the survey, indicating acute rather than chronic disease burden.^27^

We excluded observations with missing information or outlier data on age in months, HAZ score, parental height, and hemoglobin levels (eAppendix 1 in the Supplement). Our final analytic sample comprised 134 882 children and adolescents across 14 216 villages within 285 districts and 23 states and union territories in India.

### Statistical Analysis

We described the mean (for continuous variables), percentage (for categorical variables), and standard deviations of covariates in our study by child age groups and sex. We graphed linear growth plots using HAZ scores across ages 0 to 18 years, by sex, to observe whether distinct ages of growth faltering (ie, decline in HAZ score) occurred in our sample. We graphically compared HAZ score trends among children and adolescents (by sex) in villages with no open defecation with children and adolescents in villages with any prevalence of open defecation. We also compared HAZ scores by age based on the presence or absence of boiled or filtered drinking water, separately for boys and girls.

We used ordinary least-squares regression models to estimate coefficients and 95% CIs of exposures (ie, village-level open defecation, household-level access to boiled or filtered water) potentially associated with HAZ scores in children and adolescents aged 0 to 18 years, by age groups and sex. A detailed description of the regression equation appears in eAppendix 2 in the Supplement. We conducted separate regression analyses for 4 age groups, as follows: 1 year and younger, older than 1 year to 7 years, older than 7 years to 12 years, and older than 12 years to 18 years. We arrived at these age categories empirically, based on distinct inflection points in HAZ score trajectories obtained from graphical analyses reflecting periods of approximately linear growth. Within these age groups, we examined boys and girls separately given their fundamentally different growth trajectories.^40,41^ We also tested the sensitivity of analytic results to geographic location through stratified regressions by rural or urban area of residence.

We performed regression decomposition to model ideal (ie, hypothetical) scenarios, as follows: (1) open defecation changes from its sample mean to 0 (ie, elimination of open defecation), (2) access to boiled or filtered water changes from its sample mean to 1 (ie, universal access to boiled or filtered water), and (3) SES changes from its sample mean to quintile 5 (ie, highest quintile).^36,42^ We estimated separate regressions for each covariate listed above, with HAZ score as the outcome (separately for boys and girls). Using the β coefficients obtained in the regressions described above, we estimated the difference in HAZ scores with change in exposure values (from mean to ideal values, per covariate). We then graphed this difference in HAZ score (over age) for each of the 3 covariates to compare the hypothetical change in estimated HAZ score for each ideal scenario.

We conducted all analyses using Stata SE version 14.3 (StataCorp). All regression estimates were adjusted for cluster-robust standard errors. We considered a 2-sided *P* < .05 statistically significant. Data analysis was performed from June 1, 2019, to August 20, 2019.

## Results

We included 134 882 children and adolescents aged 0 to 18 years, with 70 463 (52.2%) boys and 64 419 (47.8%) girls. Table 1 shows summary statistics of our analytical sample by age group; 6631 participants (4.9%) were aged 1 year and younger, 41 244 (30.5%) were aged older than 1 year to 7 years, 40 285 (30.0%) were aged older than 7 years to 12 years, and 46 722 (34.6%) were aged older than 12 years to 18 years. Among male children and adolescents, mean (SD) HAZ score was greatest at age 1 year and younger (−1.26 [2.07] SDs) and lowest for those aged older than 12 years to 18 years (−1.86 [1.21] SDs). Similarly, among female children and adolescents, mean (SD) HAZ score was greatest for the group aged 1 year and younger (−1.09 [1.94] SDs) and lowest for those aged older than 12 years to 18 years (−1.60 [1.08] SDs). The mean (SD) of village-level open defecation was 23.6% (32.2%). A mean (SD) of 31.6% (46.5%) of households used boiled or filtered drinking water (34 145 households). A mean (SD) of 59.4% (49.1%) of households were in rural areas, and 57 065 children and adolescents (42.3%) reported living in villages with open defecation and lack of household-level access to boiled or filtered water (eTable 1 in the Supplement).

**Table 1.** Summary Statistics of Final Sample of 134 882 Children in India Who Participated in the District Level Household and Facilities Survey

Variable	Mean (SD) by age group				
	≤1 y (n = 6631)	>1 To ≤7 y (n = 41 244)	>7 To ≤12 y (n = 40 285)	>12 To ≤18 y (n = 46 722)	All age groups
Boys					
No. (%)	3564 (53.7)	21 832 (53.0)	21 331 (53.0)	23 736 (50.8)	70 463 (52.2)
Height-for-age *z* score	−1.26 (2.07)	−1.32 (1.71)	−1.38 (1.33)	−1.86 (1.21)	−1.52 (1.49)
Girls					
No. (%)	3067 (46.3)	19 412 (47.0)	18 954 (47.0)	22 986 (49.2)	64 419 (47.8)
Height-for-age *z* score	−1.09 (1.94)	−1.26 (1.68)	−1.52 (1.34)	−1.60 (1.08)	−1.45 (1.42)
All					
Height, cm					
Paternal	163.65 (7.81)	163.11 (7.61)	163.05 (7.48)	163.07 (7.35)	163.10 (7.49)
Maternal	153.43 (6.98)	153.40 (6.88)	153.78 (6.87)	153.89 (6.70)	153.65 (6.82)
Education, y					
Paternal	10.07 (3.83)	9.82 (4.00)	9.76 (4.27)	9.81 (4.50)	9.80 (4.25)
Maternal	9.97 (3.76)	9.84 (3.89)	9.74 (4.02)	9.69 (4.18)	9.78 (4.02)
Maternal age, y	26.92 (5.68)	29.72 (5.74)	36.90 (5.70)	39.23 (5.79)	34.21 (5.74)
Hemoglobin level, g/dL	9.14 (2.60)	9.96 (2.40)	10.70 (2.36)	10.87 (2.51)	10.39 (2.44)
Rural household, %	61.2 (49.7)	60.1 (48.9)	59.4 (49.1)	58.5 (49.3)	59.4 (49.1)
Village with open defecation, %	26.6 (33.8)	24.4 (32.6)	23.3 (31.9)	22.8 (31.6)	23.6 (32.2)
Boiled or filtered water, %	28.2 (45.0)	31.2 (46.3)	32.5 (46.6)	31.8 (46.6)	31.6 (46.5)

SI conversion factor: To convert hemoglobin to grams per liter, multiply by 10.0.

**Figure 1.**
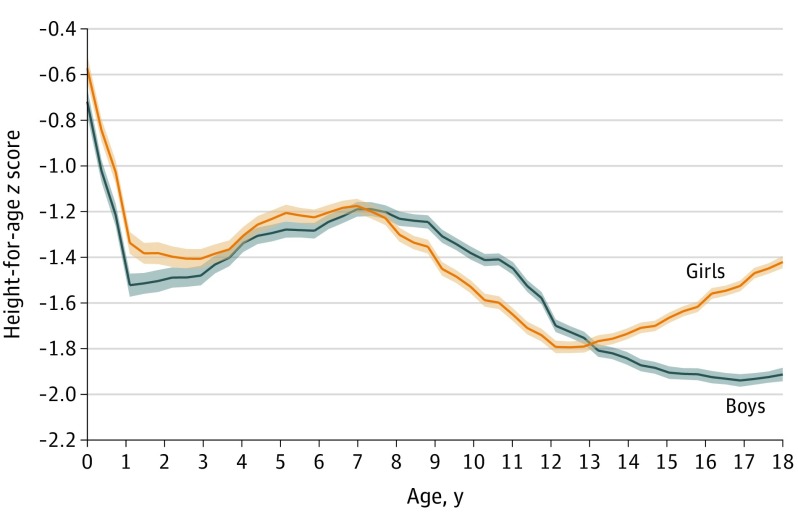
Sex-Specific Height-for-Age *z* Scores Among Children Aged 0 to 18 Years in India Height-for-age *z* scores were measured in terms of standard deviations (SDs) relative to child anthropometric reference provided by the World Health Organization.^40,41^

**Figure 2.**
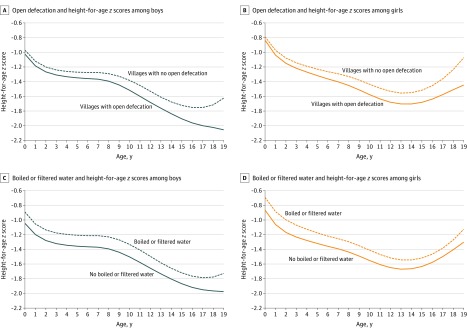
Height-for-Age *z* Scores Among Children Aged 0 to 18 Years According to Level of Open Defecation and Access to Boiled or Filtered Water Height-for-age *z* scores were measured in terms of standard deviations (SDs) relative to child anthropometric reference provided by the World Health Organization.^40,41^

Figure 1 shows trends in HAZ scores from age 0 to 18 years by sex. We observed distinct inflection points in HAZ scores among both boys and girls at ages 1, 7, and 12 years. Figure 2A and Figure 2B contrast mean HAZ score for boys and girls, respectively, (by age) between villages with no open defecation vs those with open defecation. Among boys and girls aged older than 7 years to 12 years, mean (SD) HAZ scores in villages without open defecation were −1.33 (1.37) SDs and −1.44 (1.36) SDs, respectively. In the same age group, mean (SD) HAZ scores in villages with open defecation were relatively lower, at −1.46 (1.31) SDs among boys and −1.63 (1.35) SDs among girls. Figure 2C and Figure 2D present trends in HAZ score by access to boiled or filtered drinking water. Among boys and girls aged older than 1 year to 7 years, mean (SD) HAZ scores in households with access to boiled or filtered water were −1.23 (1.69) SDs and −1.18 (1.66) SDs, respectively. In the same age group, mean (SD) HAZ scores in households without access to boiled or filtered water were relatively lower, at −1.37 (1.75) SDs among boys and −1.32 (1.73) SDs among girls. This difference persisted across all childhood ages (Figure 2).

Among boys, village-level open defecation was inversely associated with HAZ scores across all age groups (except for those aged 1 year and younger), with the largest β observed among those aged older than 1 year to 7 years (β, −0.22; 95% CI, −0.35 to −0.10), followed by those aged older than 7 years to 12 years (β, −0.15; 95% CI, −0.24 to −0.06), and those aged older than 12 to 18 years (β, −0.10; 95% CI, −0.19 to −0.01) (Table 2). Access to boiled or filtered drinking water was positively associated with HAZ scores among boys across all age groups (≤1 year: β, 0.19; 95% CI, 0.03 to 0.35; >1 to ≤7 years: β, 0.07; 95% CI, 0.00 to 0.14; >7 to ≤12 years: β, 0.08; 95% CI, 0.03 to 0.13; >12 to ≤18 years: 0.06; 95% CI, 0.01 to 0.11). Among girls, village-level open defecation was inversely associated with HAZ scores for those aged older than 7 years to 12 years (β, −0.22; 95% CI, −0.33 to −0.12) and those aged older than 12 years to 18 years (β, −0.16; 95% CI, −0.23 to −0.09) but not for other age groups. Access to boiled or filtered drinking water was positively associated with HAZ scores among girls only for those aged 1 year and younger (β, 0.26; 95% CI, 0.07 to 0.45) and those aged older than 1 year to 7 years (β, 0.07; 95% CI, 0.01 to 0.14).

**Table 2.** Regression Results Modeling Height-for-Age *z* Scores as a Function of Open Defecation and Access to Boiled or Filtered Drinking Water Among Children Aged 0 to 18 Years in India in 2013

Characteristic	β (95% CI) by age group^a^			
	≤1 y (n = 6631)	>1 To ≤7 y (n = 41 244)	>7 To ≤12 y (n = 40 285)	>12 To ≤18 y (n = 46 722)
**Boys**				
No. (%)	3564 (53.7)	21 832 (53.0)	21 331 (53.0)	23 736 (50.8)
Village open defecation, 10% increase in proportion	−0.12 (−0.41 to 0.17)	−0.22 (−0.35 to −0.10)^b^	−0.15 (−0.24 to −0.06)^b^	−0.10 (−0.19 to −0.01)^c^
Boiled or filtered drinking water, compared with untreated water	0.19 (0.03 to 0.35)^c^	0.07 (0.00 to 0.14)^c^	0.08 (0.03 to 0.13)^d^	0.06 (0.01 to 0.11)^c^
*R*^2^	0.09	0.07	0.11	0.16
**Girls**				
No. (%)	3067 (46.3)	19 412 (47.0)	18 954 (47.0)	22 986 (47.8)
Village open defecation, 10% increase in proportion	−0.25 (−0.56 to 0.07)	−0.10 (−0.23 to 0.02)	−0.22 (−0.33 to −0.12)^b^	−0.16 (−0.23 to −0.09)^b^
Boiled or filtered drinking water, compared with untreated water	0.26 (0.07 to 0.45)^d^	0.07 (0.01 to 0.14)^c^	0.03 (−0.03 to 0.09)	0.01 (−0.03 to 0.05)
*R*^2^	0.11	0.08	0.11	0.18

^a^ Models adjusted for child age, father’s height, mother’s height, household socioeconomic status, rural residence, father’s education, mother’s education, mother’s age, child’s hemoglobin level, state fixed effects and birth year fixed effects. Standard error estimates are robust and clustered at the district level.

^b^ *P* < .001.

^c^ *P* < .05.

^d^ *P* < .01.

**Figure 3.**
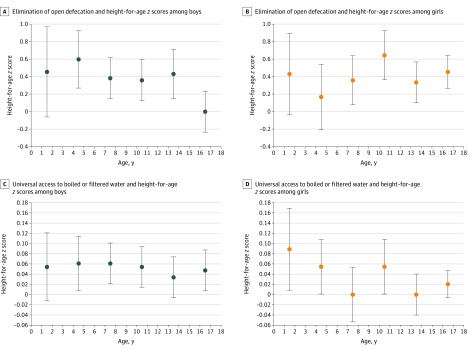
Estimated Difference in Height-for-Age *z* Score From Linear Regression–Decomposition of Open Defecation and Access to Boiled or Filtered Drinking Water for Children Aged 0 to 18 Years in India These panels show the differences in estimated height-for-age *z *score associated with change in village-level open defecation from 32.2% (sample mean) to 0% (ie, elimination of open defecation) (A, B) and change in access to boiled or filtered drinking water from 46.5% (sample mean) to 100% (ie, universal access to boiled/filtered drinking water) (C, D). Dots indicate means; whiskers, SDs.

Figure 3 shows the results of linear regression–decomposition of HAZ score with the elimination of open defecation (Figure 3A and Figure 3B) and universal access to boiled or filtered drinking water (Figure 3C and Figure 3D). Moving children from the mean level of open defecations (ie, 32%) to no open defecation corresponded with an increase in HAZ score by 0.04 SD among boys aged older than 9 years to 12 years and by 0.07 SD among girls aged older than 9 years to 12 years. For the same age group, HAZ scores increased by 0.06 SD among both boys and girls when access to boiled or filtered water increased from the sample mean (ie, 46.5%) to 100%. The eFigure in the Supplement shows regression decomposition results for change in SES from quintile 3 to 5. Moving children from mean SES (quintile 3) to high SES (quintile 5) corresponded with an increase in HAZ score by approximately 0.18 SD among boys aged older than 9 years to 12 years and by 0.03 SD among girls aged older than 9 years to 12 years. The combined (ie, additive) magnitude of HAZ score estimates associated with complete elimination of open defecation and universal access to boiled or filtered water was approximately the same as moving all households in the sample from mean SES to high SES.

The association between sanitation and HAZ score may vary by rural vs urban location. Therefore, we stratified our sample based on rural vs urban residence and reestimated the association between our outcome and exposures using methods described in eAppendix 2 in the Supplement. Results appear qualitatively similar to our original findings (eTable 2 and eTable 3 in the Supplement). However, for both boys and girls aged 0 to 18 years we observed a stronger inverse association between open defecation and HAZ scores in urban locations (boys: −0.20 SD; 95% CI, −0.30 to −0.10 SD; girls: −0.26 SD; 95% CI, −0.35 to −0.16 SD) compared with rural areas (boys: −0.15 SD; 95% CI, −0.24 to −0.07 SD; girls: −0.12 SD; 95% CI, −0.21 to −0.04 SD).

## Discussion

We examined the association between HAZ score and 2 sanitation exposures (ie, open defecation and access to boiled or filtered water) in a cross-sectional sample of 134 882 children and adolescents aged 0 to 18 years in India. We found that village-level open defecation was inversely associated with HAZ scores across most age groups, with the strongest associations during early and middle childhood in boys and middle to late childhood in girls. We also observed that household-level access to boiled or filtered drinking water was positively associated with HAZ scores across all ages for boys and during early childhood for girls. Results from linear regression–decomposition scenarios showed that HAZ score gains (ie, positive difference in HAZ estimates) under ideal sanitation conditions (ie, universal access to boiled or filtered water and elimination of open defecation) may be similar to the height advantage associated with high SES.

Among girls, access to toilets (ie, absence of open defecation) may be particularly relevant around menarche.^43,44^ Women in India experience significantly greater barriers in access to appropriate sanitation facilities.^45,46^ School absenteeism among teenage girls also increases during menstruation because of lack of toilets or sanitation infrastructure that affords privacy in schools.^47^ Whereas the examination of these mechanisms was beyond the scope of the present study, future research may examine whether the observed inverse association between village-level open defecation and HAZ scores among adolescent females arises from sociocultural barriers in access to sanitation.

We found a stronger association between sanitation-related exposures and child HAZ scores in urban compared with rural households. This difference may arise from high open defecation density (ie, number of people defecating in the open per square kilometer) in densely populated urban regions.^13^ Whereas the DLHS-4 did not provide sufficient information to calculate open defecation density, studies using other data sets in India have found that exposure to high levels of defecation density per primary sampling unit is inversely associated with height among children younger than 5 years.^13^

Recent randomized clinical trials in other LMICs have not found any association between sanitation interventions and improved linear growth in children younger than 5 years.^48,49,50,51^ These findings motivate the evaluation of such interventions on height gain from birth through late adolescence. Interventions that target behaviors associated with negative externality (eg, open defecation) may translate into improved health outcomes on achievement of a certain threshold. For instance, achievement of herd immunity through vaccination relies on immunizing a critical mass (eg, 60%-70%) of children.^52^ Reducing open defecation may correspond with improved height in children after a similar threshold of sanitation is achieved.^53^

Our study holds particular relevance in light of India’s recent national sanitation program, Swachh Bharat Mission (SBM). Launched in October 2014, SBM set the goal of providing toilets for every household and the complete elimination of open defecation by 2019. This program combined government subsidies for toilet construction with intensive behavioral messaging through multiple channels, such as radio and television, social media, cinema, and community mobilization.^54^ Thus far, more than 100 million toilets have been constructed nationwide.^55^ However, it remains unclear whether the population uses these toilets appropriately.^56^ The next phase of SBM aims to provide clean piped water to every household by the year 2024.^55^ Other public health measures, such as the recent introduction of a rotavirus vaccine in India’s universal immunization program, may augment ongoing national investments in sanitation for improved child health outcomes.^57,58^

### Strengths and Limitations

The strengths of our study include the novel examination of sanitation-related associations of growth in a large sample of children and adolescents aged 0 to 18 years using data that cover multiple geographies in India. The DLHS data series possesses strong measurement validity, shows consistency with other national surveys in India, and has been used in nearly 50 peer-reviewed publications.^59^ Currently, the absence of anthropometric data on older children and adolescents in the Demographic Health Surveys restricts research on height gain beyond age 5 years in LMICs.^60^ To our knowledge, we are the first to describe the association between poor sanitation and growth faltering across the entire span of childhood. We also incorporated strong confounders of child height, such as parental height, parental education, and household SES, as individual-level controls.^38^ Our analytic strategy also ruled out confounding from unobserved birth cohort and regional factors.

This study has limitations. The analysis was cross-sectional in nature; hence, we cannot measure exposure of older children to poor sanitation during their early childhood. Adverse growth because of prolonged exposure to poor sanitation may accrue over time and exert a cumulatively greater influence on height gain in later childhood.^26^ However, based on prior research, we assume that village-level variation in open defecation and access to boiled or filtered water does not depart starkly from historical trends, given that these variables have changed very slowly over time.^13,18^ We encourage future research to longitudinally examine changes in HAZ score following changes in sanitation. Such analyses may also include infant and young child feeding practices that may influence height gain through older ages.^61^ We also did not have information on migration patterns that may be associated with differential exposure to sanitation, and we encourage future research to examine this association. Furthermore, DLHS-4 did not include high burden Indian states (ie, Bihar, Uttar Pradesh, Madhya Pradesh, Odisha, Rajasthan, Uttarakhand, Chattisgarh, Jharkhand, and Assam), and thus, our estimates have unknown external validity to these regions.

## Conclusions

In this study, we found that open defecation and lack of access to boiled or filtered water were inversely associated with height-for-age measures among a cross-section of 134 882 children and adolescents aged 0 to 18 years in India. Our findings suggest that a reduction in open defecation and improved access to clean drinking water may benefit growth across multiple periods in childhood and adolescence.

## Notes

Supplement. **eAppendix 1. **Brief Description of Child Anthropometric Measurement and Coding of Exposure Variable in DLHS-4 Data Set **eAppendix 2. **Description of Control Variables and Equation for Regression Analysis **eFigure. **Estimated Difference in Height-for-Age *z* Scores From Linear Regression–Decomposition of Socioeconomic Status for Children Aged 0 to 18 Years in India **eTable 1. **Children Aged 0 to 18 Years Living in Villages With and Without Open Defecation and With and Without Household-Level Access to Boiled or Filtered Drinking Water **eTable 2. **Height-for-Age *z* Scores Associated With Open Defecation and Clean Drinking Water Among Boys Aged 0 to 18 Years in India in Rural and Urban Areas **eTable 3.** Height-for-Age *z* Scores Associated With Open Defecation and Clean Drinking Water Among Girls Aged 0 to 18 Years in India in Rural and Urban Areas **eReferences.**
