# Supplementary material for: Association of Poor Sanitation With Growth Measurements Among Children in India
Source: JAMA Netw Open. 2020 Apr 15;3(4):e202791. doi: 10.1001/jamanetworkopen.2020.2791 (PMC7160693; doi:10.1001/jamanetworkopen.2020.2791)
Supplement: Supplement. — eAppendix 1. Brief Description of Child Anthropometric Measurement and Coding of Exposure Variable in DLHS-4 Data Set eAppendix 2. Description of Control Variables and Equation for Regression Analysis eFigure. Estimated Difference in Height-for-Age z Scores From Linear Regression–Decomposition of Socioeconomic Status for Children Aged 0 to 18 Years in India eTable 1. Children Aged 0 to 18 Years Living in Villages With and Without Open Defecation and With and Without Household-Level Access to Boiled or Filtered Drinking Water eTable 2. Height-for-Age z Scores Associated With Open Defecation and Clean Drinking Water Among Boys Aged 0 to 18 Years in India in Rural and Urban Areas eTable 3. Height-for-Age z Scores Associated With Open Defecation and Clean Drinking Water Among Girls Aged 0 to 18 Years in India in Rural and Urban Areas eReferences. [file jamanetwopen-3-e202791-s001.pdf]

Supplementary Online Content

Chakrabarti S, Singh P, Bruckner T. Association of poor sanitation with growth measurements among children in India. *JAMA Netw Open*. 2020;3(4):e202791. doi:10.1001/jamanetworkopen.2020.2791

eAppendix 1. Brief Description of Child Anthropometric Measurement and Coding of Exposure Variable in DLHS-4 Data Set

eAppendix 2. Description of Control Variables and Equation for Regression Analysis

eFigure. Estimated Difference in Height-for-Age z Scores From Linear Regression–Decomposition of Socioeconomic Status for Children Aged 0 to 18 Years in India

eTable 1. Children Aged 0 to 18 Years Living in Villages With and Without Open Defecation and With and Without Household-Level Access to Boiled or Filtered Drinking Water

eTable 2. Height-for-Age z Scores Associated With Open Defecation and Clean Drinking Water Among Boys Aged 0 to 18 Years in India in Rural and Urban Areas

eTable3. Height-for-Age z Scores Associated With Open Defecation and Clean Drinking Water Among Girls Aged 0 to 18 Years in India in Rural and Urban Areas

eReferences.

This supplementary material has been provided by the authors to give readers additional information about their work.

© 2020 Chakrabarti S et al. *JAMA Network Open.*

eAppendix 1. Brief Description of Child Anthropometric Measurement and Coding of Exposure Variable in DLHS-4 Data Set

The data in DLHS 4 were collected from August 2012 to February 2014 and anthropometric measurements were taken for all children aged 0 to 18 years in surveyed households. In the DLHS 4 survey, child anthropometrics were measured according to the Demographic Health Survey protocols in India [1]. The Seca 874 digital scale was used to measure the weight of children and adults. The height of adults and children aged 24-59 months was measured with the Seca 213 stadiometer. The Seca 417 infantometer was used to measure the recumbent length of children under two years or less than 85 cm[2]. Fieldworkers selected for data collection of the DHLS surveys are highly qualified (minimum qualification: diploma in para-medical courses), undergo intensive pre-test training, spot checking during survey interviews and are supervised at multiple levels to maintain data fidelity[3,4].

The DLHS questionnaire asks respondents about the type of toilet facility in their household and responses are coded as 12 mutually exclusively categories[5]. Survey responses for drinking water source at the household-level are coded into 15 categories and water treatment methods to make it safe to drink are coded into 9 types [5].

Sample exclusions were made for children without valid age reported in months (N=77,247), outlier or missing data on child anthropometry (N=113,735), mother's height (N=39,919), father's height (N=64,555), and hemoglobin (N=31,967). While exclusions based on child age in months and child anthropometry are unavoidable, exclusions based on parental height and hemoglobin were deemed necessary to obtain unbiased associations between exposures and the outcomes. Specifically, the importance of parental height as a potential confounder superseded the loss of power due to smaller sample size[6]. Moreover, there were no systematic patterns observed in missing parental height or hemoglobin.

© 2020 Chakrabarti S et al. *JAMA Network Open.*

eAppendix 2. Description of Control Variables and Equation for Regression Analysis

In this study, control variables for regression analyses include household SES, parental (mother, father) height, education, age, child's age in months, child's hemoglobin level, and birth-year, state fixed effects. Birth-year fixed effects control for factors unique to each annual birth cohort which may vary over time. Inclusion of birth year fixed effects reduces omitted variable bias that arises from shared patterns over time in HAZ and other covariates. State fixed effects control for all unobserved time invariant state-level factors that may impact HAZ and other covariates. Examples of such factors include deeply ingrained dietary practices and differing state-level institutions, policies and programs that existed in 2013. Inclusion of birth-year and state fixed effects exploits the within birth year and within state variation in HAZ and sanitation in our sample.

Regression Equation

For child i, born in year t, to parent p, in household h, village v and state s we specified the following regression model, estimated using ordinary least squares.

$$\boldsymbol{HAZ_{i,t,p,h,v,s} = \beta_0 + \beta_1 OD_{v,s} + \beta_2 DW_{h,v,s} + \beta_3 HH_{h,v,s} + \beta_4 Parent_{p,h,v,s} + \beta_5 Child_{i,t,p,h,v,s} + \beta_6 Birthyear_t + \beta_7 State_s + \varepsilon_{i,t,p,h,v,s}}$$

Where

HAZ represents the height-for-age z-score of children aged 0-19 years

β_0 is the intercept

$\boldsymbol{OD_{v,s}}$ the proportion of households reporting open defecation in a village $\boldsymbol{DW_{h,v,s}}$ is household-level indicator of access to boiled/filtered drinking water

$\boldsymbol{HH_{h,v,s}}$ is a vector of household SES and residence variables

$\boldsymbol{Parent_{p,h,v,s}}$ is a vector of parental height, education and age

$\boldsymbol{Child_{i,t,p,h,v,s}}$ is the vector of child age and hemoglobin level

$\boldsymbol{Birthyear_t}$ are birth-year fixed effects

$\boldsymbol{State_s}$ are state fixed effects

$\boldsymbol{\varepsilon_{i,t,p,h,v,s}}$ is the heteroscedasticity robust standard error corrected for clustering at the district-level

© 2020 Chakrabarti S et al. *JAMA Network Open.*

eFigure. Estimated Difference in Height-for-Age *z* Scores From Linear Regression–Decomposition of Socioeconomic Status for Children Aged 0 to 18 Years in India

Panel A: Males

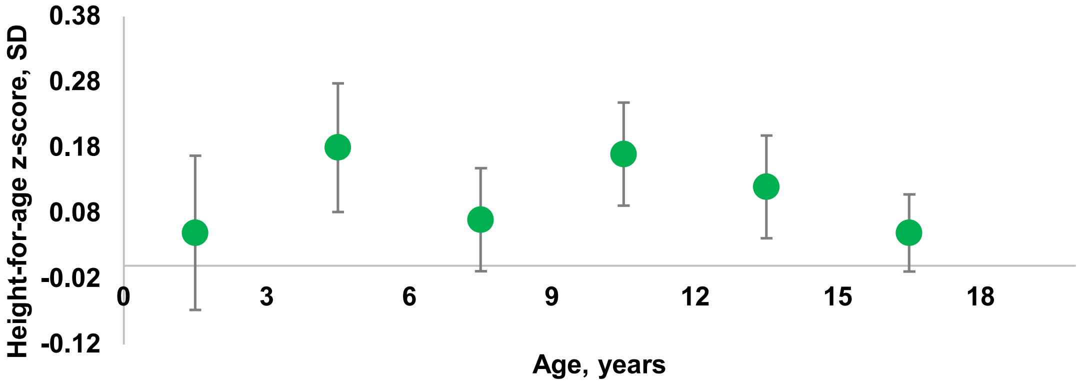

Panel B: Females

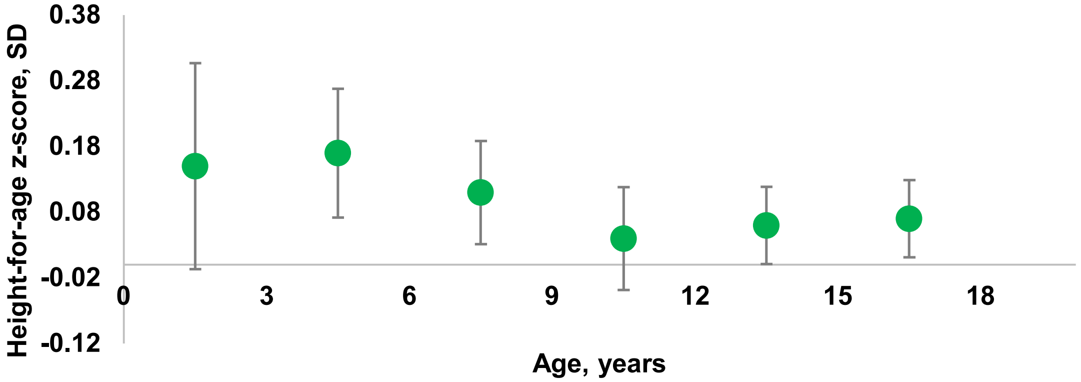

eFigure1 Legend: eFigure1 shows difference in estimated HAZ (with 95% CIs) associated with change in household-level SES from Quintile 3 (sample mean) to Quintile 5 (i.e. highest SES level). HAZ differences are estimated over narrow age intervals (≤ 3 years, >3 to ≤6 years, >6 to ≤ 9 years, >9 to ≤ 12 years, >12 to ≤ 15 years, >15 to ≤ 18 years). These age intervals were selected to approximate continuous age with equal spacing between successive age groups. HAZ estimates (by age and sex group) present the association between child height and (hypothetical) ‘ideal’ scenario wherein all households in the study sample moved to highest SES quintile.

© 2020 Chakrabarti S et al. *JAMA Network Open.*

eTable 1. Children Aged 0 to 18 Years Living in Villages With and Without Open Defecation and With and Without Household-Level Access to Boiled or Filtered Drinking Water

	Household-level access to boiled/filtered drinking water	
	**Yes (N, %)**	**No (N, %)**
**Village-level open defecation: Yes (N, %)**	11,119 (8%)	57,065 (42.3%)
**Village-level open defecation: No (N, %)**	31,592 (23.4%)	35,186 (26.1%)

© 2020 Chakrabarti S et al. *JAMA Network Open.*

eTable 2. Height-for-Age *z* Scores Associated With Open Defecation and Clean Drinking Water Among Boys Aged 0 to 18 Years in India in Rural and Urban Areas

**Outcome: Height-for-age z-score, SD**	**≤1 year**		**>1 to ≤7 years**		**>7 to ≤12 years**		**>12 to ≤18 years**		**All age groups (0 to 18 years)**	
	**Coefficient**	**95% CI**	**Coefficient**	**95% CI**	**Coefficient**	**95% CI**	**Coefficient**	**95% CI**	**Coefficient**	**95% CI**
Rural										
Village open defecation, proportion	-0.2	[-0.59,0.19]	-0.19*	[-0.35,-0.03]	-0.14*	[-0.25,-0.02]	-0.13*	[-0.23,-0.02]	-0.15***	[-0.24,-0.07]
Clean (boiled/treated) drinking water (reference = untreated water)	0.05	[-0.19,0.29]	0.05	[-0.03,0.14]	0.06	[-0.00,0.12]	0.05	[-0.02,0.11]	0.06*	[0.01,0.10]
**N**	**2174**		**13186**		**12563**		**13847**		**41770**	
Urban										
Village open defecation, proportion	-0.24	[-0.74,0.25]	-0.34**	[-0.54,-0.13]	-0.21**	[-0.35,-0.06]	-0.03	[-0.18,0.11]	-0.20***	[-0.30,-0.10]
Clean (boiled/treated) drinking water (reference = untreated water)	0.31*	[0.07,0.56]	0.09	[-0.01,0.19]	0.09*	[0.01,0.17]	0.06	[-0.01,0.13]	0.09**	[0.04,0.15]
**N**	**1390**		**8646**		**8768**		**9889**		**28693**	

Standard error estimates are robust and clustered at the district-level.
Models adjusted for child age, father's height, mother's height, household socio-economic status, rural residence, father's education, mother's education, mother's age, child's hemoglobin level, state fixed-effects and birth-year fixed effects.
* $p < 0.05$, ** $p < 0.01$, *** $p < 0.001$

© 2020 Chakrabarti S et al. *JAMA Network Open.*

eTable3. Height-for-Age *z* Scores Associated With Open Defecation and Clean Drinking Water Among Girls Aged 0 to 18 Years in India in Rural and Urban Areas

Outcome: Height-for-age z-score, SD	≤1 year		>1 to ≤7 years		>7 to ≤12 years		>12 to ≤18 years		All age groups (0 to 18 years)	
	Coefficient	95% CI	Coefficient	95% CI	Coefficient	Coefficient	95% CI	Coefficient	Coefficient	95% CI
Rural										
Village open defecation, proportion	-0.01	[-0.41,0.39]	-0.03	[-0.19,0.13]	-0.20**	[-0.34,-0.06]	-0.17***	[-0.26,-0.08]	-0.12**	[-0.21,-0.04]
Clean (boiled/treated) drinking water (reference = untreated water)	0.26*	[0.04,0.49]	0.05	[-0.03,0.14]	0.07	[-0.01,0.15]	0.01	[-0.04,0.06]	0.06*	[0.01,0.10]
N	1887		11602		11351		13491		38331	
Urban										
Village open defecation, proportion	-0.66*	[-1.26,-0.06]	-0.22*	[-0.42,-0.02]	-0.31***	[-0.46,-0.15]	-0.13*	[-0.25,-0.01]	-0.26***	[-0.35,-0.16]
Clean (boiled/treated) drinking water (reference = untreated water)	0.3	[-0.01,0.60]	0.08	[-0.01,0.17]	-0.02	[-0.10,0.07]	0.01	[-0.05,0.06]	0.04	[-0.01,0.08]
N	1180		7810		7603		9495		26088	

Standard error estimates are robust and clustered at the district-level.
Models adjusted for child age, father's height, mother's height, household socio-economic status, rural residence, father's education, mother's education, mother's age, child's hemoglobin level, state fixed-effects and birth-year fixed effects.
* $p < 0.05$, ** $p < 0.01$, *** $p < 0.001$

© 2020 Chakrabarti S et al. *JAMA Network Open.*

eReferences.

1. Dandona R, Pandey A, Dandona L. A review of national health surveys in India. *Bull World Health Organ*. 2016. doi:10.2471/blt.15.158493
2. Ministry of Health and Family Welfare Government of India. *National Family Health Survey-4, 2015-2016, India Report*.; 2017.
3. International Institute of Population Sciences - Ministry of Health and Family Welfare (MoHFW) - Government of India. *Supervisor's and Editor's Manual: District Level Household Survey-3 under Reproductive and Child Health Project.*; 2008. http://rchiips.org/pdf/rch3/Manual/3_Supervisors_manual_DLHS- 3 .pdf.
4. International Institute of Population Sciences - Ministry of Health and Family Welfare (MoHFW) - Government of India. District Level Household Survey-4 Pre-Bid Conference. In: *Pre-Bid Conference*. ; 2012. http://iipsindia.org/pdf/pre bid conference combine PP.pdf.
5. International Institute of Population Sciences - Ministry of Health and Family Welfare (MoHFW) - Government of India. District Level Household Survey 2008-2009 (DLHS-4): Household Questionnaire-Under Reproductive and Child Health Project (RCH). 2008. http://rchiips.org/pdf/rch3/Questionaire/1_Household Questionaire.pdf.
6. Özaltin E, Hill K, Subramanian S V. Association of maternal stature with offspring mortality, underweight, and stunting in low- to middle-income countries. *JAMA - J Am Med Assoc*. 2010;303(15):1507-1516. doi:10.1001/jama.2010.450

© 2020 Chakrabarti S et al. *JAMA Network Open.*
